# Stratifications and foliations in phase portraits of gene network models

**DOI:** 10.18699/VJGB-22-91

**Published:** 2022-12

**Authors:** V.P. Golubyatnikov, A.A. Akinshin, N.B. Ayupova, L.S. Minushkina

**Affiliations:** Sobolev Institute of Mathematics of the Siberian Branch of the Russian Academy of Sciences, Novosibirsk, Russia Novosibirsk State University, Novosibirsk, Russia; Huawei Russian Research Institute, St. Petersburg, Russia; Sobolev Institute of Mathematics of the Siberian Branch of the Russian Academy of Sciences, Novosibirsk, Russia Novosibirsk State University, Novosibirsk, Russia; Novosibirsk State University, Novosibirsk, Russia

**Keywords:** oscillations, positive and negative feedbacks, gene network models, phase portraits, invariant domains and surfaces, invariant foliations, Poincaré map, Grobman–Hartman theorem, Frobenius–Perron theorem, осцилляции, положительные и отрицательные связи, модели генных сетей, фазовые портреты, инвариантные области и поверхности, инвариантные слоения, отображение Пуанкаре, теорема Гробмана–Хартмана, теорема Фробениуса–Перрона.

## Abstract

Periodic processes of gene network functioning are described with good precision by periodic trajectories (limit cycles) of multidimensional systems of kinetic-type differential equations. In the literature, such systems are often called dynamical, they are composed according to schemes of positive and negative feedback between components of these networks. The variables in these equations describe concentrations of these components as functions of time. In the preparation of numerical experiments with such mathematical models, it is useful to start with studies of qualitative behavior of ensembles of trajectories of the corresponding dynamical systems, in particular, to estimate the highest likelihood domain of the initial data, to solve inverse problems of parameter identification, to list the equilibrium points and their characteristics, to localize cycles in the phase portraits, to construct stratification of the phase portraits to subdomains with different qualities of trajectory behavior, etc. Such an à priori geometric analysis of the dynamical systems is quite analogous to the basic section “Investigation of functions and plot of their graphs” of Calculus, where the methods of qualitative studies of shapes of curves determined by equations are exposed. In the present paper, we construct ensembles of trajectories in phase portraits of some dynamical systems. These ensembles are 2-dimensional surfaces invariant with respect to shifts along the trajectories. This is analogous to classical construction in analytic mechanics, i. e. the level surfaces of motion integrals (energy, kinetic moment, etc.). Such surfaces compose foliations in phase portraits of dynamical systems of Hamiltonian mechanics. In contrast with this classical mechanical case, the foliations considered in this paper have singularities: all their leaves have a non-empty intersection, they contain limit cycles on their boundaries. Description of the phase portraits of these systems at the level of their stratifications, and that of ensembles of trajectories allows one to construct more realistic gene network models on the basis of methods of statistical physics and the theory of stochastic differential equations.

## Abstract (ru)

Периодические процессы функционирования широкого класса генных сетей с хорошей точностью описываются предельными циклами многомерных систем дифференциальных уравнений кинетического типа. Такие системы, часто называемые в литературе динамическими, составляются по схемам положительных и отрицательных связей между компонентами моделируемых сетей. Искомые функции в уравнениях описывают зависимость от времени концентраций этих компонент. При планировании вычислительных экспериментов с подобными математическими моделями полезно предварительно описать качественное поведение ансамблей траекторий соответствующих динамических систем, в частности оценить области максимального правдоподобия начальных данных, исследовать обратные задачи идентификации параметров, особые точки этих систем, локализовать в фазовых портретах положение циклов, в том числе предельных стратифицировать фазовые портреты на подобласти с качественно различным поведением траекторий и т. п. Такой априорный геометрический анализ рассматриваемых моделей генных сетей полностью аналоги- чен хрестоматийному разделу начальных курсов математики «Исследование функций и построение графи- ков», в котором описываются методы наглядного представления поведения кривых, определяемых уравне- ниями. В настоящей статье в фазовых портретах динамических систем, моделирующих функционирование кольцевых генных сетей, конструируются двумерные поверхности, инвариантные относительно сдвигов вдоль траекторий, – ансамбли траекторий. Просматривается естественная аналогия с классической кон- струкцией аналитической механики – с поверхностями уровня интегралов движения (энергия, импульс и др.). Такие поверхности образуют слоения в фазовых портретах динамических систем гамильтоновой меха- ники. В отличие от задач механики, для рассматриваемых нами моделей генных сетей слоения, образуемые инвариантными поверхностями, имеют особенности, все их слои содержат на своих границах предельные циклы. Описание фазовых портретов динамических систем в терминах их стратификаций и ансамблей их траекторий позволит строить более реалистичные модели генных сетей с использованием аппарата стати- стической физики и теории стохастических дифференциальных уравнений.

## Introduction

At present time, investigation of questions of existence of periodic trajectories (cycles) in phase portraits of systems of non-linear differential equations simulating functioning of various natural processes is carried out in most fields of applied mathematics. Detection of such cycles, their localization in the phase portraits, description of their characteristics, such as stability, (non)uniqueness, etc. have a long history (Poincaré, 1892). These problems have generated a whole range of research directions in pure mathematics: qualitative theory of differential equations, theory of dynamics systems, etc., which in turn have a great impact on corresponding applied disciplines. At their junction, the famous 16-th Hilbert’s problem, and the “center-focus” problem, related to seemingly just a pictorial case of two differential equations with two unknown functions of one variable (time) have appeared.

Here, in the present paper, we study systems of kinetic equations of higher dimensions, considered as functioning of circular gene networks models:

**Formula. 1.**
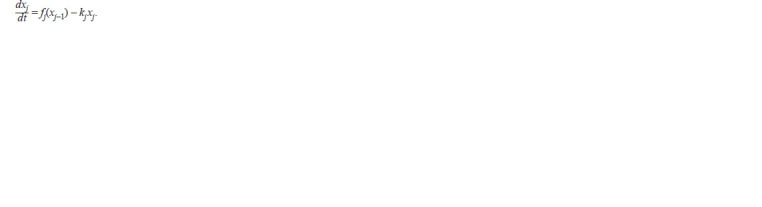
Formula. 1.

It is assumed here and below that j = 1, 2, …, n; n ≥ 3, and that j – 1 = n, if j = 1. In all these equations, non-negative functions xj (t) denote concentrations of species in the gene networks, and positive coefficients kj characterize the rates of their degradations (Likhoshvai et al., 2020).

Consider the system (1) in the vector form dX dt = F(X ), where the vector-function X(t) is defined by its coordinate functions xj(t). The divergence of this vector-field F(X ) is constant and negative: div F(X ) ≡ –k1 – k2 – … – kn < 0.

It is well-known (Arnold, 1989) that in this case, n-dimensional volume of any finite domain in the phase portrait decreases exponentially during the shifts of its points along trajectories of the system (1) as t grows. This does not mean that each such domain collapses to a point. For the dynamical systems considered here, these limit sets are two-dimensional invariant surfaces in their n-dimensional phase portraits.

We call the dynamical system (1) block-linear if for all j each function fj which describes the rate of synthesis of the j-th component of the gene network is a step-function (threshold function) fj( y) ≡ Lj( y) = kj aj, if 0 ≤ y ≤ 1; Lj( y) ≡ 0, if y > 1; or fj ( y) ≡ Γj ( y) ≡ 0, if 0 ≤ y ≤ 1; Γj ( y) ≡ kj aj, if y > 1.

Here, aj are some positive constants. Decreasing functions Lj describe negative feedbacks in the gene network and increasing functions Γj correspond to positive feedbacks.

For one particular case kj = 1 for all j, investigation of cycles of similar block-linear systems was realized in (Glass, Pasternack, 1978; Akinshin et al., 2013; Ayupova, Golubyatnikov, 2014; Golubyatnikov, Gradov, 2021). Under the same assumptions, questions of existence of cycles in smooth analogues of these systems were studied in (Elowitz, Leibler, 2000; Glyzin et al., 2016; Kolesov et al., 2016) in the cases when these systems are symmetric with respect to cyclic permutations of pairs of the variables xj.

In recent publications (Golubyatnikov, Ivanov, 2018; Golubyatnikov, Minushkina, 2019, 2020; Likhoshvai et al., 2020; Ivanov, 2022), existence, uniqueness, and stability of the cycles of block-linear dynamical systems of some different dimensions with arbitrary positive coefficients kj were proved with the help of stratification of phase portraits to subdomains according to behavior of trajectories. It was shown there that these phase portraits contain cycles if and only if aj > 1 for all j and that the parallelepiped Qn = [0, a1] × [0, a2] × … × [0, an] in the positive octant of the space ℝn is a positively invariant domain of the dynamical system (1). This means that trajectories of all points of the domain Qn do not leave it and that all cycles of the system (1) are contained in the interior of Qn. We consider below the dynamical systems of the type (1) in the case aj > 1 for all j only. Physical interpretation of this condition means that the maximal rate of synthesis of any component of the gene network exceeds that of its degra-dation.

We decompose the domain Qn by the planes xj = 1 to 2n smaller parallelepipeds, which we call blocks and enumerate by binary multi-indices: {ε1 ε2 … εn}= I1(ε1) × I2(ε2) × … × In(εn). Here, each index εj equals 0 or 1, and Ij(0) = [0, 1], Ij(1) = = (1, aj]. Let E be the common point of all these blocks (all its coordinates equal one). In each of these blocks, the equations of the system (1) take the simplest linear form dxj dt = kj (xj – aj (1 – εj–1)),

and solution to the Cauchy problem for this system has a simple representation

**Formula. 2.**
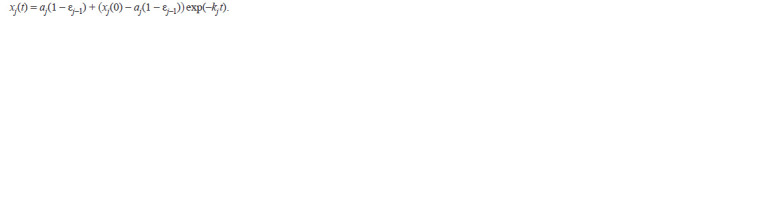
Formula. 2.

In the present paper, for some low dimensional block-linear dynamical systems considered as models of gene networks functioning, we study the behavior of ensembles of their trajectories and show the existence of families of two-dimensional surfaces that are invariant with respect to shifts along trajectories of these systems and contain their cycles. This makes the qualitative analysis of trajectory behavior and interpretation of numerical experiments with these models much simpler.

## Three-dimensional dynamical system

In the papers (Golubyatnikov et al., 2018; Golubyatnikov, Ivanov, 2018), we considered a 3D block-linear dynamic system:

**Formula. 3.**
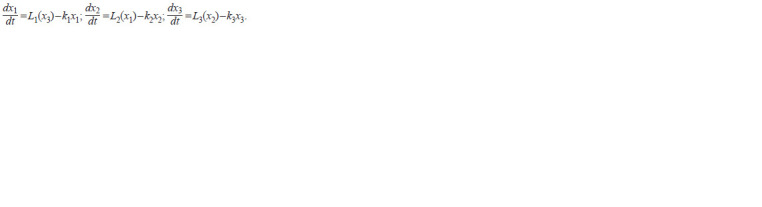
Formula. 3.

Trajectories of all points of the block {001} pass through six blocks of decomposition of the domain Q3 from block to block according to arrows of the following diagram only:

**Formula. 4.**
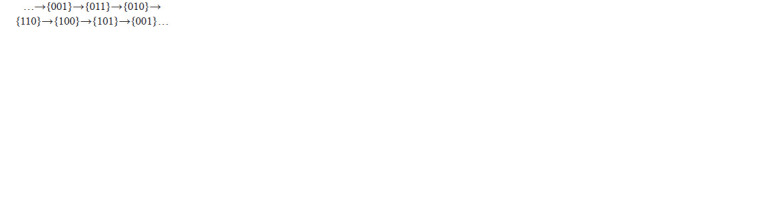
Formula. 4.

Denote by W 3 1 a union of blocks listed in the diagram, this is a positive invariant domain of the system (3), its interior is homeomorphic to torus. Note that trajectories of points of two blocks, {000} and {111}, eventually leave them in the invariant domain W 3 1 and further stay there. Thus, cycles of the system (3) do not intersect these two blocks (Golubyatnikov et al., 2018). Stratification of phase portrait of the system (3) consists of two parts: the domain W 3 1 and the union of two blocks, {000}, {111}.

Consider a two-dimensional face F0 = {001}∩{011} which separates the blocks {001} and {011} as well as other faces Fm which separate incident blocks of the diagram (4):F1 = {011}∩{010}, F2 = {010}∩{110}, F3 = {110}∩{100},… F5 = {101}∩{001}.

On the face F0, let us introduce a coordinate system (w1; w2) with the origin at the point E3 = (1; 1; 1) such that coordinates w1, w2 of all points of this face are non-negative: w1 = 1 – x2; w2 = x3 – 1. Let the Poincaré map be written by equation Ψ(w1; w2) = (ψ1(w1; w2); ψ2(w1; w2)).The main technical result of the papers (Golubyatnikov et al., 2018; Golubyatnikov, Ivanov, 2018) is the following

Lemma 1: а) the Poincaré map is monotonic: if for points A(v1; v2) and B(w1; w2) relations v1 < w1 and v2 < w2, are satisfied then ψ1(v1; v2) < ψ1(w1; w2) and ψ2(v1; v2) < ψ2(w1; w2). For this partial order relation, we use a notation: A B, Ψ(A) Ψ(B); b) if w1 and w2 are sufficiently small then w1 < ψ1(w1; w2) and w2 < ψ2(w1; w2), i. e., B Ψ(B); c) at each point of the face F0, the first derivatives of the coordinate functions ψ1 and ψ2 are strictly positive and their second derivatives are strictly negative.

This implies that the Poincaré map Ψ:F0→F0 has two fixed points exactly; one of them is the point E3 which lies at the boundary of F0 and the other one, denoted by P* , is contained in the interior of the face F0 (Golubyatnikov, Ivanov, 2018). Trajectory of the point P* returns to this point after transition through the blocks of the diagram (4) and, therefore, it is a cycle. Since the map Ψ has just one nontrivial fixed point P* , the system (3) does not have any other cycles

In the same paper, for the fixed points E3 and P* of the Poincaré map, Jacobian matrices J2(E3) and J2(P*) were calculated and it was shown that the eigenvalues λ1(P*), λ2(P*) of the matrix J2(P*) are different, positive and do not exceed one, which means exponential stability of the cycle of the system (3). We denote this cycle discovered in (Golubyatnikov, Ivanov, 2018) by ℂ3. Lemma 1 also implies that both these Jacobian matrices are positive, so it is possible to use the Frobenius–Perron theorem (Gantmacher, 1959) in our studies.

Note that the determinant of Jacobian matrix J2(E3) is equal to one and for its eigenvalues λ1(E3), λ2(E3), relations λ1(E3) > 1 > λ2(E3) > 0 are true. So, for the map Ψ, hypothesis of Grobman–Hartman theorem (Hartman, 1964) is fulfilled. This implies that in a sufficiently small neighborhood U(E3) ⸦ F0 of the point E3, the Poincaré map is linearized by some continuous (in general terms, non-smooth) change of variables (w1; w2) (u1; u2). In such a coordinate system, Ψ(u1; u2) = = (λ1(E3) · u1; λ2(E3) · u2).

For sufficiently small ε > 0, we denote by T 2 ε ⸦ U(E3) a triangle 0 ≤ u1 + u2 < ε with one vertex at the point E3 and let F0 be a truncated face F0\T 2 ε

Choose two segments [0, α1] and [0, α0] ⸦ [0, α1] in this neighborhood so that α1 = λ 1(E3) · α0. Let N1 and N0, respectively, be the right endpoints of these segments, then Ψ([0, α0]) = [0, α1] and Ψ(N0) = N1; in the original coordinate system (w1; w2), the segments [0, α0] and [0, α1] are represented by arcs D0 ⸦ D1 with a common endpoint E3. Consider action of iterations of the Poincaré map to these arcs:Ψ(D0) = D1 ⸦ D2 := Ψ(D1) ⸦ D3 := Ψ(D2) ⸦ D4 …

The union D* of infinite sequence of mutually embedded arcs Dk is a continuous monotonic arc connecting the points E3 and P* ; after transition along arrows of the diagram (4), trajectories of points of D* return to this arc: the semi-interval D1\D0 passes to semi-interval D2\D1 which passes in turn to D3\D2, etc. Thus, trajectories of points of the arc D* generate an invariant (non-smooth) surface Σ2 bounded by the cycle ℂ3 in the invariant domain W 3 1 ⸦ Q3. By the construction, this surface contains the point E3.

Starting such constructions of small segments [N0, N1] in a neighborhood U(E3) with points N0 which do not lie on the axis E3u1 and considering the images of these segments under iterations of the Poincaré map Ψ, we obtain a family of continuous monotonic arcs which leave the neighborhood U(E3) and do not contain the point E3. For each pair of points N0, N1 ⸦ U(E3)\ E3u1 such that Ψ(N0) = N1, the sequence Nk = Ψ(Nk–1) tends monotonically to the fixed point P* of the Poincaré map Ψ (Golubyatnikov et al., 2018). Here, each segment [N0, N1] generates, as above, a monotonic arc D* (N0) being invariant with respect to the Poincaré map. Trajectories of points of such an arc, in their turn, form an invariant 2D surface Σ2(N0) which intersects the surface Σ2 by the cycle ℂ3 exactly.

In a similar way, one can construct invariant surfaces which do not intersect the neighborhood U(E3) in the domain W 3 1. Let U(P* ) ⸦ F0 be a neighborhood of the nontrivial fixed point P* , where the map Ψ can be linearized. We save the notations (u1; u2) for these linearized coordinates. For sufficiently small ε > 0, the Poincaré map transforms the ellipsis S 1 1 ⸦ U(P* ) with equation λ1(P*)u 2 1 + λ2(P*)u 2 2 = ε2 to the circle S 1 0 with the equation u 2 1 + u 2 2 = ε2. Let I1(M0) be a segment which joins the point M1 S 1 1 with its image M0 = Ψ(M1) S 1 0 . All such segments are contained in U(P* ) in the ring between S 1 0 and S 1 1 . Each of these segments generates a sequence of continuous arcs Dk(M0), they are invariant with respect to the Poincaré map, and Ψ(Dk(M0)) = Dk–1(M0). For each of these arcs, trajectories of its points generate in W 3 1 an invariant surface bounded by the cycle ℂ3.

Theorem 1. There exists two-dimensional invariant foliation in the invariant domain W 3 1 of the dynamical system (3); its leaves fill W 3 1 and contain the cycle ℂ3 on their boundaries. One of these leaves contains the point E3.

## Four-dimensional dynamical system

Recently, in the papers (Ayupova, Golubyatnikov, 2019; Golubyatnikov, Minushkina, 2021), we considered a fourdimensional block-linear system

**Formula. 5.**
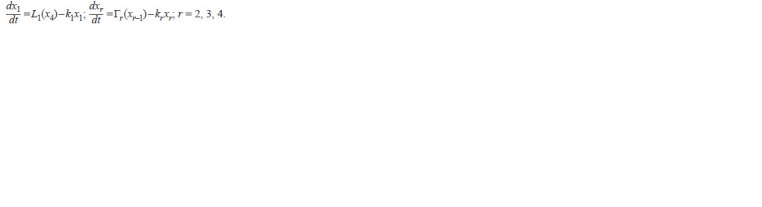
Formula. 5.

In particular case, when kj = 1 for all j, questions of existence, uniqueness, and stability of cycles of such systems were studied in (Glass, Pasternack, 1978). Smooth analogues of similar systems were considered in (Hastings et al., 1977; Mallet-Paret, Smith, 1990).

An invariant domain Q4 of the system (5) is decomposed by hyperplanes xj = 1 to 16 blocks {ε1 ε2 ε3 ε4}. Blocks of this decomposition listed in the following diagram form an invariant subdomain W 4 1 in the phase portrait of (5)

**Formula. 6.**
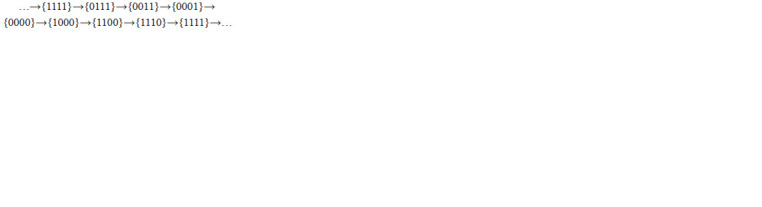
Formula. 6.

The arrows of this diagram show the only possible direction of trajectory transition from one block to another. The subdomain W 4 1 is one of two parts of stratification of the phase portrait of the system (5). For each block not listed here, trajectories of its points can pass to three adjacent blocks, two of them are contained in W 4 1, and one is in Q4\W 4 1 (this is the second part of the stratification mentioned above). Algorithms of construction of such diagrams for the systems of arbitrary dimensions, both smooth and blocks-linear, are described in (Kazantsev, 2015; Kirillova, Minushkina, 2019).

As in previous sections, let us denote by 0 an intersection of two adjacent blocks {1111}∩{0111} in the diagram (6). After eight steps according to its arrows under shifts along trajectories, all points of this three-dimensional face return to 0. Let Ψ4: 0 → 0 be a corresponding Poincaré map, T 3 ε ⸦ U(E4) be a pyramid 0 ≤ u1 + u2 + u3 < ε with the vertex at the point E4 = (1; 1; 1; 1), and 0 be a truncated face 0\ T 3 ε .

In the paper (Golubyatnikov, Minushkina, 2021), it was shown that all statements of Lemma 1 are true for the map Ψ4, thus, this map has two fixed points exactly: E4 and the point П* which is contained in the interior of the face 0. This means that the invariant domain W 4 1 of the system (5) contains one cycle exactly, let us denote it by ℂ4. The following results were also established there

Lemma 2: a) the Jacobi matrices J3(E4) and J3(П*) and their determinants are positive; b) det J3(E4) = λ1(E4) · λ2(E4) · λ3(E4) = 1; c) magnitudes of eigenvalues of the matrix J3(П*) are less than one.

This implies the exponential stability of the cycle ℂ4 and possibility of linearization of the Poincaré map Ψ4 in some small neighborhood U(П*) of its fixed point П*. According to the Frobenius–Perron theorem, one of the eigenvalues of the matrix J3(П*) is positive and greater than the magnitudes of the remaining eigenvalues. The same applies to the eigenvalues of the matrix J3(E4). Let us enumerate the eigenvalues of Jacobi matrices in order of decreasing of their absolute values: λ1 > |λ2| ≥ |λ3|. Let (u1; u2; u3) be the coordinates where Ψ4 is linear Φ(u1; u2; u3) = (λ1(Π*) · u1; λ2(Π*) · u2; λ3(Π*) · u3).

As in the case of the system (3), for a sufficiently small ε > 0, the Poincaré map translates the ellipsoid S 2 1 with the equation λ1(Π*)u 2 1 + |λ2(Π*)|u 2 2 + |λ3(Π*)|u 2 3 = ε2 to the sphere S 2 0 with the equation u 2 1 + u 2 2 + u 2 3 = ε2.

Theorem 2. If aj > 1 for all j = 1, 2, 3, 4, and the Jacobi matrix J3(E4) of the Poincaré map does not have eigenvalues with unit module then there exists an invariant foliation in the domain W 4 1; its leaves fill this invariant domain and contain the cycle ℂ4. One of these leaves contains the point E4.

## Dynamical systems of higher dimensions

In the papers (Gaidov, Golubyatnikov, 2014; Ayupova, Golubyatnikov, 2021), we considered a five-dimensional block linear dynamical system

**Formula. 7.**
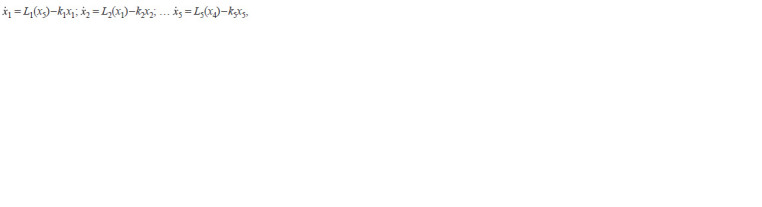
Formula. 7.

for which, as in previous sections, an invariant domain Q5 = = [0, a1] × [0, a2] × … × [0, a5] and its decomposition to blocks by the hyperplanes xj = 1 were constructed. Ten blocks of this decomposition form a stratum W 5 1 ⸦ Q5 which is invariant with respect to shifts along trajectories of the system (7) passing through the blocks according to arrows of a cyclic diagram, similar to (4) and (6): …→{10101}→{00101}→{01101}→{01001}→ {01011}→{01010}→{11010}→{10010}→ {10110}→{10100}→{10101}→…

Points of the four-dimensional face F 4 0 = {10101}∩{00101} under shifts along their trajectories after ten steps along the arrows of the diagram return to the face F 4 0 .

For such a Poincaré map Ψ5:F 4 0 →F 4 0 , an analogue of Lemma 1 implies that the face F 4 0 contains two fixed points of this map exactly: the point E5 = (1; 1; 1; 1; 1) and a point П5 * in the interior of this face. The domain W 5 1 contains one cycle exactly. Let us denote it by ℂ5. This cycle is stable and passes through the point П5 * (Ayupova, Golubyatnikov, 2021).

As in previous sections, an analogue of Lemma 2 holds: Jacobi matrices J4(E5), J4(П5 *) and their determinants are positive, det J4(E5) = 1.

The magnitudes of eigenvalues of the matrix J4(П5 *) do not exceed one. In the case when these Jacobi matrices do not have any eigenvalues modulo equal to 1, construction of the invariant surface Σ2 ⸦ W 5 1 with the boundary ℂ5 and an invariant foliation in the domain W 5 1 is carried out exactly in the same way as above.

In the paper (Golubyatnikov, Gradov, 2021), conditions under which a non-invariant stratum Q5 \ (W 5 1 {00000} {11111}) of the phase portrait of the five-dimensional system (7) contains one more of its cycle were established.

Similar constructions can be done for a block-linear analogue of the six-dimensional Elowitz–Leibler system (Elowitz, Leibler, 2000) studied in (Minushkina, 2021; Golubyatnikov, Minushkina, 2022)

**Formula. 8.**
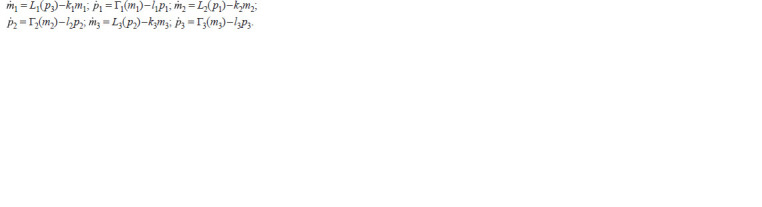
Formula. 8.

Here, the variables mj and pj and denote concentrations of three mRNAs and proteins TetR, Lacl and λcl, corresponding to them (Elowitz, Leibler, 2000; Kolesov et al., 2016).

An invariant domain Q6 = Π 3 j =1[0, aj] × [0, bj], where bj are the maximum values of step functions Гj divided by the coefficients lj, j = 1, 2, 3, is decomposed by six hyperplanes mj = 1, pj = 1, j = 1, 2, 3, to 64 blocks which form a stratification of Q6 to three subdomains, W 6 1 , W 6 3 , and W 6 5 , with different qualitative trajectory behavior.

The domain W 6 5 consists of 12 blocks, from which trajectories can transit to 5 adjacent blocks. In the symmetric case when kj = lj = 1, there are no cycles in this subdomain. However, W 6 5 contains a two-dimensional invariant surface consisting of piecewise linear trajectories attracting by the point E6 = (1; 1; 1; 1; 1; 1) in a spiral way.

In the domain W 6 1 formed by 12 blocks, from which trajectories can enter one adjacent block only, the Poincaré map contains a unique non-trivial fixed point П6 *, the trajectory of which is a stable limit cycle for all trajectories in this domain (Golubyatnikov, Minushkina, 2022).

In the domain W 6 3 which consists of 40 blocks, state transition diagram has a more complicated combinatorial structure. At present time, transitions of trajectories from one block to another in this subdomain have not been studied completely yet.

For smooth analogues of the dynamical system (8), the uniqueness of equilibrium point was established in (Ayupova et al., 2017). As in the case of block linear systems, hyperplanes passing through the equilibrium point and being parallel to coordinate ones decompose the invariant domain Q6 to 64 blocks. If a linearization matrix of such smooth system in its equilibrium point has eigenvalues with positive and negative real parts and does not have any imaginary eigenvalues then the invariant domain W 6 1 contains a cycle ℂ6 of this sytem (Ayupova et al., 2017). In the paper (Kirillova, 2020), conditions of existence of an invariant surface bounded by the cycle ℂ6 in the domain W 6 1 were obtained.

## Results of numerical experiments

The lefthand part of Figure shows 100 trajectories of the dynamical system (3). Each of these trajectories is contained in a corresponding leaf of the foliation in W 3 1 near the invariant surface Σ2. The values of parameters of this system are: k1 = 0.4; k2 = 0.3; k3 = 0.6; a1 = 1.3; a2 = 1.4; a3 = 1.7. The ini-tial data are chosen in a random way in a rectangular neighborhood of the point E3. The righthand part of this Figure shows results of similar experiments with a smooth analogue of the system (3): dx dt = 10 1+ z3 – x; dy dt = 10 1+ x3 – y; dz dt = 10 1+ y3 – z. Here, one can clearly see its invariant surface.

It was shown in (Golubyatnikov et al., 2018; Ayupova, Golubyatnikov, 2021; Golubyatnikov, Minushkina, 2021; Minushkina, 2021) that trajectories of block-linear dynamical systems (3), (5), (7), (8) are piecewise smooth, the discontinuities of their derivatives are located on the planes xj = 1, this is clearly seen on the left part of Figure

**Fig. 1.**
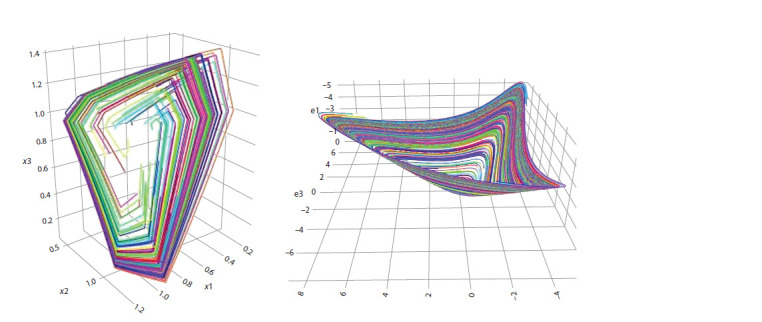
Results of numerical experiments with trajectories of the 3D systems.

In order to perform numerical simulations of trajectories of (3), we have developed a software project using the R programming language (https://www.r-project.org/) and the Shiny package (https://shiny.rstudio.com/). The source code is available on GitHub: https://github.com/AndreyAkinshin/pwLLL.

The simulations are performed in the cloud; the results are described at https://aakinshin.net/posts/dscs2/. The library ggplot (https://ggplot2.tidyverse.org/) is used here, as well as the package deSolve (http://desolve.r-forge.r-project.org/) that contains integration routines previously used to simulate other systems of gene networks. The user interface allows one to specify all parameters of the system (3).

## Conclusion

In this paper, we have described a construction of invariant foliations, i. e. the families of invariant two-dimensional surfaces in phase portraits of low-dimensional block-linear models of circular gene networks. It was shown that on each leaf of these foliations, trajectories of all its points are repelled by the boundary of the central part of the phase portrait and they are attracted by the limit cycle which describes an oscillating functioning of the corresponding gene network. Theorem 1 is illustrated by numerical experiments

For the kinetic dynamical systems under consideration, the leaves of invariant foliations in the phase portraits play the role of level surfaces of collections of motion integrals studied in classical mechanics (Poincaré, 1892; Arnold, 1989). Reduction of dimensions of invariant subsets in the phase portraits allows us to give a digestible description of trajectories behavior and, in particular, simplifies considerably numerical experiments with such gene networks models (Likhoshvai et al., 2020). Construction of the foliations mentioned above and investigation of their geometric properties can be useful in studies of dynamical characteristics of more complicated models of gene networks functioning when a description of a big system is given on the basis of known results on its subsystems which have a simpler structure.

## Conflict of interest

The authors declare no conflict of interest.
